# A systematic review of validated screening tools for anxiety disorders and PTSD in low to middle income countries

**DOI:** 10.1186/s12888-020-02753-3

**Published:** 2020-06-30

**Authors:** Anisa Y. Mughal, Jackson Devadas, Eric Ardman, Brooke Levis, Vivian F. Go, Bradley N. Gaynes

**Affiliations:** 1 The University of Pittsburgh School of Medicine, 3550 Terrace Street, Pittsburgh, PA 15213 USA; 2 Department of Health Behavior, Gillings School of Global Public Health, University of North Carolina at Chapel Hill, 135 Dauer Dr, Chapel Hill, NC 27599 USA; 3 University of Miami Miller School of Medicine, 1600 NW 10th Ave #1140, Miami, FL 33136 USA; 4 Department of Epidemiology, Biostatistics and Occupational Health, McGill University, Montreal, Quebec H3A 1A2 Canada; 5 Centre for Prognosis Research, School of Primary, Community and Social Care, Keele University, Staffordshire, ST5 5BG UK; 6 Department of Psychiatry, University of North Carolina School of Medicine, 101 Manning Dr, Chapel Hill, NC 27514 USA

**Keywords:** Anxiety, Post-traumatic stress disorder, Screening tool, Validation, Low-to-middle income countries

## Abstract

**Background:**

Anxiety and post-traumatic stress disorder (PTSD) contribute significantly to disability adjusted life years in low- to middle-income countries (LMICs). Screening has been proposed to improve identification and management of these disorders, but little is known about the validity of screening tools for these disorders. We conducted a systematic review of validated screening tools for detecting anxiety and PTSD in LMICs.

**Methods:**

MEDLINE, EMBASE, Global Health and PsychINFO were searched (inception-April 22, 2020). Eligible studies (1) screened for anxiety disorders and/or PTSD; (2) reported sensitivity and specificity for a given cut-off value; (3) were conducted in LMICs; and (4) compared screening results to diagnostic classifications based on a reference standard. Screening tool, cut-off, disorder, region, country, and clinical population were extracted for each study, and we assessed study quality. Accuracy results were organized based on screening tool, cut-off, and specific disorder. Accuracy estimates for the same cut-off for the same screening tool and disorder were combined via meta-analysis.

**Results:**

Of 6322 unique citations identified, 58 articles including 77 screening tools were included. There were 46, 19 and 12 validations for anxiety, PTSD, and combined depression and anxiety, respectively. Continentally, Asia had the most validations (35). Regionally, South Asia (11) had the most validations, followed by South Africa (10) and West Asia (9). The Kessler-10 (7) and the Generalized Anxiety Disorder-7 item scale (GAD-7) (6) were the most commonly validated tools for anxiety disorders, while the Harvard Trauma Questionnaire (3) and Posttraumatic Diagnostic Scale (3) were the most commonly validated tools for PTSD. Most studies (29) had the lowest quality rating (unblinded). Due to incomplete reporting, we could meta-analyze results from only two studies, which involved the GAD-7 (cut-off ≥10, pooled sensitivity = 76%, pooled specificity = 64%).

**Conclusion:**

Use of brief screening instruments can bring much needed attention and research opportunities to various at-risk LMIC populations. However, many have been validated in inadequately designed studies, precluding any general recommendation for specific tools in LMICs. Locally validated screening tools for anxiety and PTSD need further evaluation in well-designed studies to assess whether they can improve the detection and management of these common disorders.

**Trial registration:**

PROSPERO registry number CRD42019121794.

## Background

Mental health disorders, including anxiety and post-traumatic stress disorder (PTSD) are among the leading contributors to global disability adjusted life years, comprising five of the top twenty contributing disorders [1]. The World Health Organization International Classification of Disease (ICD-11) defines anxiety as a disorder in which there is an extreme and excessive focus on an “anticipated threat” and defines PTSD as a disorder that results from exposure to one or more “horrific events”, both of whose symptoms include apprehension, motor tension and autonomic overactivity [2]. In 2017, it was estimated that over 264 million people experienced an anxiety disorder, with the global prevalence for both anxiety disorders and PTSD ranging from 2.5 to 7% by country [2–4]. Both anxiety and PTSD are widespread common mental disorders (CMDs) that have been shown to cause significant negative health outcomes within various populations and contribute to a large portion of the global disease burden [5, 6]. There are noteworthy discrepancies in quality of life between people diagnosed with anxiety and/or PTSD and those who are not diagnosed with either, such as increased years lived with disability and decreased life expectancy [7–9]. Additionally, there is evidence suggesting that the presence of an anxiety disorder or PTSD increases the likelihood of comorbidity with other severe health conditions, such as major depressive disorder and substance use disorder [10, 11].

Anxiety and PTSD in low to middle income countries (LMICs) are highly prevalent and require further study given that access to care is hindered by availability and stigma [12–14]. Prevalence of these disorders is higher within LMICs; roughly 83% of people with mental illnesses globally are living within LMICs [15]. In many LMICs, there is no robust mental healthcare system in place and the number of mental health professionals is sparse [16]. Assessment and diagnosis of psychiatric illnesses thus often falls to primary care and general practitioners who have little training in mental health [16]. Use of brief screening tools have been proposed as a way to improve identification and management of mental health problems, and may be useful in LMICs, especially among populations with elevated risk (e.g., pregnant women, refugees/displaced persons, and youth) within LMIC communities [17–19].

Despite multiple screening instruments for CMDs, there are significantly fewer screening instruments for anxiety and PTSD that have been validated in LMIC populations. Screening instruments that have been validated exclusively in high-income countries may not perform equivalently in LMIC populations, as anxiety and PTSD often present differently in different cultural contexts. For example, in sub-Saharan Africa, anxiety and PTSD are described through somatic symptoms as well as spiritual descriptions [20]. Furthermore, differences in clinical presentation may render screening tools less accurate in LMICs. Thus, optimum cut-off scores validated in high income populations may not apply in LMIC populations. For instance, in a sample of 75 participants from Tajikistan [21], the optimal cut-off of 1.88 for the Harvard Trauma Questionnaire (HTQ), a measure of PTSD, was substantially lower than the standard cut-off score of 2.5 that has been recommended in previous studies in high-income countries [22]. Failure to apply suitable cut-off scores may lead to an imbalance of positive and negative screening results. If chosen cutoffs are too high, actual cases of anxiety and PTSD may not reach the threshold for further assessment and diagnosis; thus, cases will be missed. Conversely, if chosen cutoffs are too low, there may a very large number of positive screens requiring substantial resources for further assessment, and healthcare systems may not be able to manage the load.

Although there has been an increasing interest in studying mental health within LMICs, there are still large gaps related to screening tools to assess mental health disorders, especially anxiety and PTSD. The most recent systematic review investigating screening tools for CMDs in LMICs was published in 2016 [23]. Of the 273 validations included, 236 were validated tools for CMDs or depressive disorders while only 24 and 13 validated tools for anxiety and PTSD, respectively. Therefore, the objective of this study was to conduct a systematic review of screening tools for anxiety and PTSD within LMIC populations.

## Methods

Aim: To validate screening tools for anxiety disorders and PTSD in LMICs.

We published a study protocol in advance in the PROSPERO registry (CRD42019121794).

### Search strategy and study selection

**Fig. 1:**
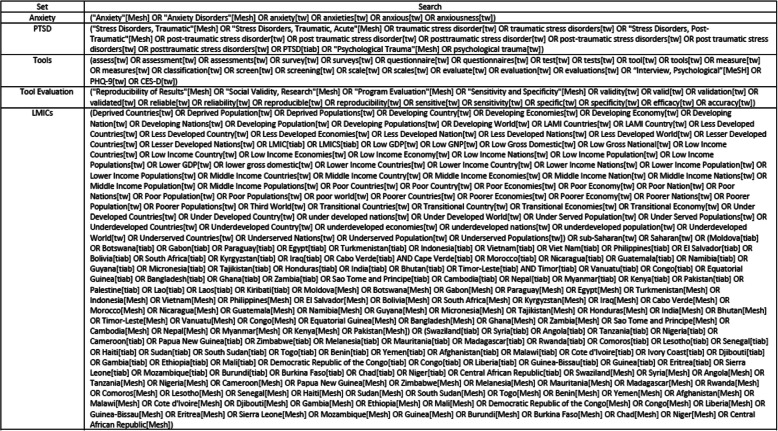
Search strategy

We systematically searched four databases (MEDLINE, EMBASE, Global Health and PsychINFO) from inception to April 22, 2020 (see Fig. 1).

### Inclusion criteria

Our eligibility criteria required that studies: (1) screen specifically for anxiety (generalized anxiety disorder or anxiety disorders not otherwise specified) and/or PTSD; (2) provide estimates of sensitivity and specificity for a given cut-off value for one of the included disorders; (3) were conducted in a LMIC (based on the World Bank Classification) [24]; and (4) compare screening results to a validated reference standard. Reference standards included unstructured clinical diagnostic interviews as well as structured clinical interviews including the Mini International Neuropsychiatric Interview (MINI and MINI-KID) [25], Structured Clinical Interview for DSM (SCID, SCID-1 and NetSCID) [26, 27], Composite International Diagnostic Interview (CIDI and CIDI-PHCV) [28], Clinical Interview Schedule-Revised (CIS-R) [29], Psychiatric Assessment Schedule (PAS) [30], Kiddie Schedule for Affective Disorders and Schizophrenia (K-SADS and K-SADS-PL) [31] and Clinician-Administered PTSD Scale (CAPS and CAPS-5) [32, 33]. LMIC populations residing in a LMIC at the time of study were included. No search restrictions were put on age, gender or comorbidities.

### Exclusion criteria

We excluded papers that did not report sensitivity, specificity and cut-off value; that were not published in English; and that involved populations originally from an LMIC residing outside a LMIC at the time of the study. Persons from an LMIC residing in another LMIC at the time of the study were included (e.g., refugee populations and displaced persons).

### Literature review

Abstracts returned from the search were reviewed separately by two independent reviewers for inclusion, with any discrepancies resolved by discussion and use of a third senior reviewer as needed. For abstracts meeting inclusion criteria, full-text articles were retrieved and reviewed by two separate reviewers for final inclusion, with discrepancies resolved by discussion and use of a third senior reviewer as needed. We also searched the reference lists of relevant systematic reviews for additional articles to add to our full-text review.

### Quality appraisal

**Fig. 2:**
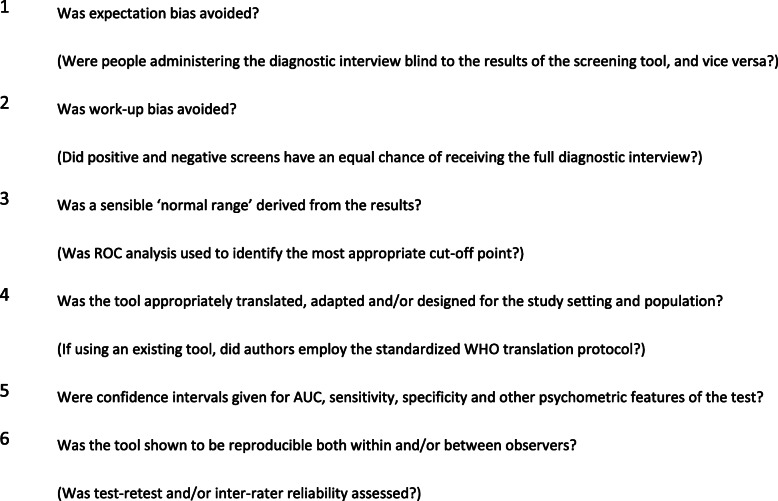
A modified Greenhalgh’s ten item checklist, adapted from Ali et al. [23]

To assess study quality, we used a modified version of Greenhalgh’s ten item checklist previously used in a study by Ali et al. [23] Elements of the quality checklist are provided in Fig. 2. Credit was given for translation if a previously validated translated version of the tool or reference standard was used, or if the tool was administered in English. Studies of ‘very good’ quality fulfilled all the quality criteria. Studies deemed ‘good’ quality fulfilled criteria 1 through 3 in addition to at least one other criterion from 4 to 5. ‘Fair’ quality studies did not avoid work-up bias and ‘acceptable’ quality studies did not perform receiver operating characteristic curve (ROC) analysis to determine a normal range from the results. ‘Unblinded’ studies include studies that reported the interviewers were not blinded to the screening results; if the study did not specify whether the screening tool administrators and interviewers were blinded to each other’s results, we considered it unblinded but clarified this designation was unconfirmed.

### Data abstraction and analysis

Numerical data was abstracted by one reviewer and checked by a separate reviewer to ensure quality extraction. Data abstraction sheets included extraction of the screening tool and disorder, number of participants, DSM version, screening tool administrator, language, region, population study characteristics and age, country, gold standard, area under the curve (AUC), cut-off score, sensitivity and specificity. If multiple screening tools and/or cut-offs were used, data was extracted for each cutoff, for each tool, separately. If values were split by population, the value most representative of the total was chosen (e.g., community values for data split by hospital inpatient unit). If multiple cut-offs were given without AUC, we extracted the set of values for the cutoff that maximized Youden’s J [34]. Results were presented separately by disorder, screening tool and cut-off value. As anxiety and depression were combined in many screening tools, a third category of mixed anxiety and depression was included.

For validations of screening tools for the same disorder that used identical cut-off values, bivariate random-effects meta-analytic models were fitted to provide estimates of pooled sensitivity and specificity for the cut-off value.

## Results

### Study selection

**Fig. 3:**
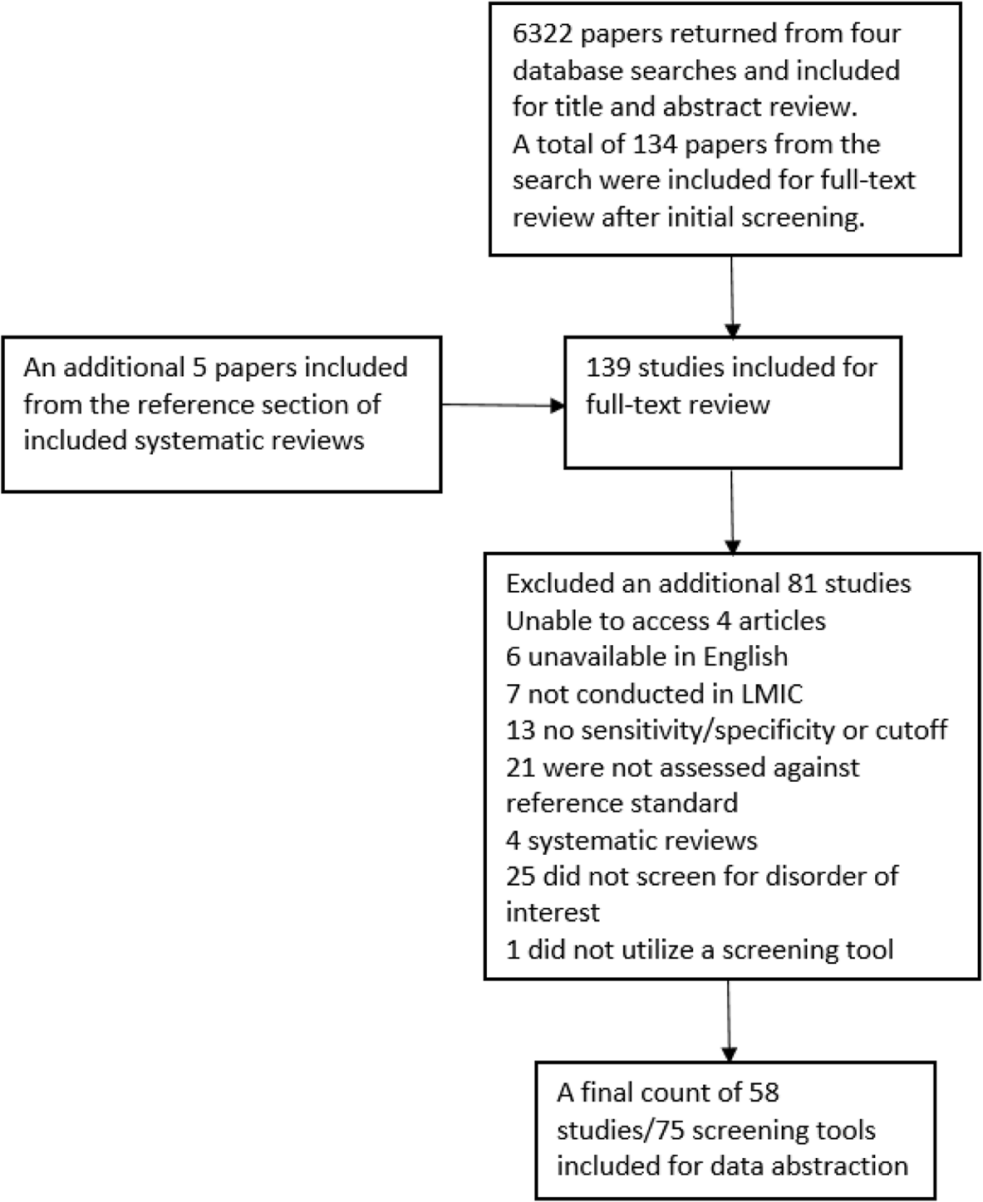
Flow chart of study selection

Of 6322 unique citations identified from the database search, 6188 were excluded after title and abstract review and five additional papers from the reference lists of relevant systematic reviews were added. Of 140 included for full-text review, 81 were excluded, leaving 59 eligible articles inclusive of 77 screening tools (see Fig. 3). The most common reasons for exclusion were not screening for the disorder of interest, not comparing to a gold standard, and failing to provide either sensitivity/specificity data or a threshold for screening.

### Quality appraisal

Two studies met all the criteria of the modified Greenhalgh’s ten item checklist and deemed ‘very good’ quality while 20 studies were deemed to be ‘good’ quality, due to lack of reporting the confidence intervals for sensitivity, specificity or AUC. Two studies were ‘fair’ quality for not avoiding work-up bias and five were deemed ‘acceptable’ for failing to perform ROC analysis. A total of 29 studies were labelled ‘unblinded’ for failing to specify if they blinded the researchers or for explicitly stating they were not blinded (see Table 1).

**Table 1:** Quality rating statistics

Quality Rating	Number of Studies
Very good	2
Good	20
Fair	2
Acceptable	5
Unblinded	29
Total	58

### Description of included studies

The final 59 studies selected included a total of 77 screening tools. There were 46 validations of screening tools for anxiety disorders, 19 for PTSD and 12 for anxiety and depression (see Table 2).

**Table 2:** Screening tool validation by disorder category

Disorder Category	Specific disorders	Total
Anxiety Disorders	Generalized Anxiety Disorder	46
	Panic Disorder	
	Social Anxiety Disorder	
	Anxiety Disorder NOS	
PTSD	PTSD	19
Anxiety and Depression	Generalized Anxiety Disorder	12
	Major Depressive Disorder	
**Total**		**77**

A minority of studies accounted for children and adolescent validations (10) despite a relatively young demographic present in LMICs [35]. The majority of validations studied adults (36), with a select few including adolescents and adults (6) (see Table 3). Particularly well-represented groups included the general population and clinical outpatients (13), perinatal populations (6), psychiatric patients (7) and those with another psychiatric comorbidity (7) (see Table 3). Of the 19 validations for PTSD, only four studied children and adolescents.

**Table 3:** Distribution by age a population characteristic

Population Descriptors		Number of Studies
**Adults (36)**	Outpatients	**5**
	General Population	**7**
	HIV	**4**
	Psychiatric patients	**7**
	Conflict area/refugee	**4**
	Other or unspecified	**9**
**Perinatal (6)**	HIV	**1**
	Other	**5**
**Adolescents and Adults (6)**	Survivors of natural disaster	**2**
	Other	**4**
**Children and/or Adolescents (10)**	Psychiatric Patient	**2**
	Survivor of natural disaster	**2**
	Other	**6**

The majority of screening tool validations were in Asia (35) followed by Africa (20), the Americas (5) and Europe (1) (see Table 4). The best represented regions include South and West Asia, as well as South and East Africa, with a noticeable gap in Middle and Northern Africa. There were no studies from the Oceanic region.

**Table 4:** Number of Studies by Region and Country

Continent	Region	Country (Number of Studies)	LMICs with no studies
Africa (20)	North	None	6 (Sudan, Algeria, Egypt, Libya, Morocco, Tunisia)
	Middle	None	9 (Angola, Cameroon, Central African Republic, Chad, Congo, Democratic Republic of the Congo, Equatorial Guinea, Gabon, Sao Tome and Principe)
	East (8)	Zimbabwe (2), Somalia (1), Uganda (1), Burundi (1), Tanzania (1), Zambia (1), Ethiopia (1)	10 (Comoros, Djibouti, Eritrea, Kenya, Madagascar, Malawi, Mauritius, Mozambique, Rwanda, South Sudan)
	West (2)	Nigeria (2)	14 (Benin, Burkina Faso, Cabo Verde, Cote dIvoire, Gambia, Ghana, Guinea-Bissau, Liberia, Mali, Mauritania, Niger, Senegal, Sierra Leone, Togo)
	South (10)	South Africa (10)	4 (Botswana, Lesotho, Namibia, Swaziland)
Asia (35)	East (7)	China (7)	2 (North Korea, Mongolia)
	South (11)	Pakistan (2), India (3), Nepal (3), Afghanistan (1), Iran (2)	4 (Bangladesh, Bhutan, Maldives, Sri Lanka)
	South East (7)	Vietnam (3), Malaysia (2), Indonesia (1), Thailand (1)	4 (Cambodia, Laos, Philippines, Timor-Leste)
	West (9)	Kuwait (1), Lebanon (3), Turkey (4), Iraq (1)	7 (Armenia, Azerbaijan, Georgia, Jordan, Palestine, Syria, Yemen)
	Central (1)	Tajikistan (1)	4 (Kazakhstan, Kyrgyzstan, Turkmenistan, Uzbekistan)
America (5)	South (4)	Brazil (2), Peru (2)	6 (Bolivia, Colombia, Ecuador, Guyana, Paraguay, Suriname)
	Central (1)	Mexico (1)	7 (Belize, Costa Rica, El Salvador, Guatemala, Honduras, Nicaragua, Panama)
	Caribbean	None	6 (Cuba, Dominica, Dominican Republic, Grenada, Haiti, Jamaica)
Europe (1)	Southern (1)	Bosnia and Herzegovina (1)	4 (Albania, Macedonia, Montenegro, Serbia)
	Eastern	None	5 (Belarus, Bulgaria, Moldova, Romania, Ukraine)
Oceania		None	2 (Melanesia, Micronesia)
Total (61^a^)			

^a^The country total is 61 instead of 58 as one study [36] involved four countries (Mexico, China, Brazil and Pakistan)

The most commonly used tools to screen for generalized anxiety disorder were the Kessler-10 (K-10) and the Generalized Anxiety Disorder-7 item scale (GAD-7), totaling seven and six validations respectively. The Hopkins Symptom Checklist-25 item scale (HSCL-25), Hospital Anxiety and Depression Scale (HADS) and Hospital Anxiety and Depression Scale anxiety subscale (HADS-A) were validated almost equally while the majority of tools only had one validation (see Table 5). PTSD had far fewer validations (19) with a wide range of tools receiving between one and three validations, similar to the screening tools validated for both anxiety and depression.

**Table 5:** Screening Tool by Disorder and Number of Validations

Disorder	Screening Tool	Number of Validations
Anxiety disorders	HADS-A	3
	HADS	3
	DASS-A	1
	Zung SAS	2
	STAI	1
	EPDS	2
	HAM-A	1
	K10	7
	K6	3
	PHQ-4	1
	GAD-7	6
	HDRS	1
	HSCL-25	4
	MINI-SPIN	1
	PHC	1
	GHQ-12	2
	SCARED/SCARED-C/−P	1/1/1
	PASS	1
	RCADS-GAD scale	1
	BAI	2
	Total	46
PTSD	HTQ/−R	1
	HTQ	3
	K10	2
	PDS	3
	PCL-C/−5	2/2
	CPSS	2
	TSSC	1
	UCLA PTSD Index	1
	PTSD Screening Tool	2
	Total	19
Anxiety and Depression	HSCL-25	2
	Independently developed (Zambia)	1
	YSR	1
	HADS	1
	AKUADS	1
	SRQ-20	1
	AYMH	1
	HEI	1
	K10/K6	1/1
	PHQ-4	1
	Total	12

*Abbreviations*: *HADS* Hospital Anxiety and Depression Scale, *HADS-A* Hospital Anxiety and Depression Scale Anxiety subscale, *DASS* Depression Anxiety Stress Scales, *Zung SAS* Zung Self-Rating Anxiety Scale, *STAI* State Trait Anxiety Inventory, *EPDS* Edinburgh Postnatal Depression Scale, *HAM-A* Hamilton Anxiety Rating Scale, *K10/K6* Kessler 10/6, *GAD* Generalized Anxiety Test, *HDRS* Hamilton Depression Rating Scale, *HSCL* Hopkins Symptom Checklist, *MINI-SPIN* Mini-Social Phobia Inventory, *PHC* Primary Health Care Screening Tool, *GHC* General Health Questionnaire, *SCARED* Screen for Child Anxiety Related Disorders, *PASS* Perinatal Anxiety Screening Scale, *RCADS* Revised Children’s Anxiety and Depression Scales, *BAI* Beck Anxiety Inventory, *HTQ* Harvard Trauma Questionnaire, *PDS* Posttraumatic Diagnostic Scale, *PCL-C* PTSD Checklist-Clinician Version, *PHQ-4* Patient Health Questionnaire, *CPSS* Child PTSD Symptom Scale, *TSSC* Traumatic Stress Symptom Scale, *YSR* Youth Self-Report, *AKUADS* Aga Khan University Anxiety and Depression, *SRQ* Self-Reporting Questionnaire, *AYMH* Arab Youth Mental Health Scale, *HEI* Huaxi Emotional-Distress Index

Each included study is listed in Table 6 by region, screening tool and study quality with the respective sensitivity, specificity and cut-off for each disorder. Continentally, Asia had the most validations (35) and the majority of studies were considered unblinded (29). Due to incomplete reporting, we could meta-analyze results from only two studies, which involved the GAD-7; using a cut-off ≥10; sensitivity = 76%, specificity = 64%.

**Table 6:** Included studies listed by continent, sub-region, screening tool/disorder and quality

Author (year)	Screening tool/disorder	Gold Standard	Subregion	Country	Population	Study Quality	No. Participants	Prevalence (%)	DSM Version	AUC	Cut-Off Score (≥)	Sensitivity (%)	Specificity (%)
**Africa**													
Ventevogel et al. (2014) [37]	CPSS/PTSD	K-SADS-PL	Africa East	Burundi	Children aged 10–15	good	65	23	DSM 4	0.78	26	71	83
Chibanda et al. (2016) [38]	GAD-7/GAD	SCID	Africa East	Zimbabwe	Adults except pregnant women	good	264	3	DSM 4	0.9	10	89	73
Kaaya et al. (2002) [39]	HSCL-25/Anxiety and depression	SCID	Africa East	Tanzania	Pregnant women with HIV	good	903 (100 for SCID)	3.3	DSM 4	0.86	1.06	89	80
Verhey et al. (2018) [40]	PCL-5/PTSD	CAPS-5	Africa East	Zimbabwe	Adults except perinatal women	very good	204	19.6	DSM 5	0.78	33	74.5	70.6
Odenwald et al. (2007) [41]	PDS/PTSD	CIDI	Africa East	Somalia	Patients with trauma exposure	good	135 (62 for CIDI)	16.1	DSM 4	0.874	14	90	79
Ertl et al. (2011) [42]	PDS/PTSD	CAPS	Africa East	Uganda	Adults and adolescents aged 12–25	good	68	32.4	DSM 4	0.79	16	82	70
Mbewe et al. (2013) [43]	self-made/Anxiety and depression	Interview	Africa East	Zambia	Adults with epilepsy	good	575	53.7	DSM 4	x	17	56.5	68.1
Geibel et al. (2016) [44]	YSR/anxiety and depression	Interview	Africa East	Ethiopia	Vulnerable teens assisted by two aid organizations	good	134	64.6	DSM 4	0.729	6.5	75	63.1
Saal (2019) [45]	Beck Anxiety Inventory/GAD	SCID	Africa South	South Africa	Adults undergoing HIV testing	unblinded*	500	3.4	DSM 5	0.86	21.5	82	80
van Heyningen et al. (2018) [46]	EPDS/anxiety	MINI	Africa South	South Africa	Adult women in the antenatal period	unblinded*	376	23	DSM 4	0.69	5	67	59
Marsay et al. (2017) [47]	EPDS/anxiety	NetSCID	Africa South	South Africa	Adult women pregnant for 22–28 weeks	unblinded*	145	14.5	DSM 5	x	7	54.8	81.6
van Heyningen et al. (2018) [46]	GAD-2/anxiety	MINI	Africa South	South Africa	Adult women in the antenatal period	unblinded*	376	23	DSM 4	0.73	2	64	74
Seedat et al. (2007) [48]	HADS-A/anxiety	MINI	Africa South	South Africa	Adult schizophrenic patients	unblinded	70	22.9	DSM 4	x	11	37.5	72.2
Seedat et al. (2007) [48]	HAM-A/anxiety	MINI	Africa South	South Africa	Adult schizophrenic patients	unblinded	70	22.9	DSM 4	x	22	31.3	90.7
Myer et al. (2008) [49]	HTQ/PTSD	MINI	Africa South	South Africa	HIV-positive adults	good	465	5	DSM 4	0.74	62	74	70
Spies et al. (2009) [50]	K-10/Agoraphobia	MINI	Africa South	South Africa	HIV-positive adults	unblinded*	429	18.4	DSM 7	0.69	26	65	67
van Heyningen et al. (2018) [46]	K10/anxiety	MINI	Africa South	South Africa	Adult women in the antenatal period	unblinded*	376	23	DSM 4	0.77	11	76	70
Andersen et al. (2011) [51]	K-10/Anxiety and Depression	CIDI	Africa South	South Africa	Adults	unblinded	4077	x	DSM 4	0.73	16	70	67
Spies et al. (2009) [52]	K10/GAD	MINI	Africa South	South Africa	HIV-positive adults	unblinded*	429	18.4	DSM 4	0.78	30	72	80
Spies et al. (2009) [50]	K-10/GAD	MINI	Africa South	South Africa	HIV-positive adults	unblinded*	429	18.4	x	0.78	30	72	80
Spies et al. (2009) [50]	K-10/Panic disorder	MINI	Africa South	South Africa	HIV-positive adults	unblinded*	429	15.3	DSM 6	0.77	28	76	73
Spies et al. (2009) [52]	K-10/PTSD	MINI	Africa South	South Africa	HIV-positive adults	unblinded*	429	21.5	DSM 8	0.77	29	75	78
Spies et al. (2009) [50]	K-10/PTSD	MINI	Africa South	South Africa	HIV-positive adults	unblinded*	429	21.5	x	0.77	29	75	78
Spies et al. (2009) [50]	K-10/Social anxiety	MINI	Africa South	South Africa	HIV-positive adults	unblinded*	429	12.3	DSM 5	0.9	30	92	80
van Heyningen et al. (2018) [46]	K6/anxiety	MINI	Africa South	South Africa	Adult women in the antenatal period	unblinded*	376	23	DSM 4	0.77	8	69	76
Andersen et al. (2011) [51]	K-6/Anxiety and Depression	CIDI	Africa South	South Africa	Adults	unblinded	4077	x	DSM 4	0.72	10	70	62
Martin et al. (2009) [53]	PDS/PTSD	CIDI	Africa South	South Africa	HIV-positive adults	unblinded	85	x	DSM 4	0.74	15	68.6	65
van der Westhuizen (2016) [54]	SRQ-20/Anxiety/Depression	MINI	Africa South	South Africa	Adults with assault-related injury or accidents	unblinded*	200	x	ICD 10	0.87	5	83.3	76
Seedat et al. (2007) [48]	STAI/anxiety	MINI	Africa South	South Africa	Adult schizophrenic patients	unblinded	70	22.9	DSM 4	x	40	75	48.1
Makanjuola et al. (2014) [55]	GHQ-12/anxiety	CIDI	Africa West	Nigeria	Adult patients of general practices	unblinded	1590	x	DSM 4	0.61	3	59	63.3
Abiodun et al. (1994) [56]	HADS/Anxiety and Depression	Interview	Africa West	Nigeria	Adult patients in non-psychiatric wards and community	unblinded*	1078	Various†	ICD 9	x	8	87.5	90.6
Makanjuola et al. (2014) [55]	K6/anxiety	CIDI	Africa West	Nigeria	Adult patients of general practices	unblinded	1590	x	DSM 4	0.58	4	65	55
**Asia**													
Hollander et al. (2007) [21]	HSCL-25/anxiety	Interview	Asia Central	Tajikistan	Adult patients at outpatient clinics	acceptable	75	x	DSM 4	x	1.6	84	60
Hollander et al. (2007) [21]	HTQ-R/PTSD	Interview	Asia Central	Tajikistan	Adult patients at outpatient clinics	acceptable	75	x	DSM 4	x	1.73	97	65
Tong et al. (2016) [57]	GAD-7/Generalized anxiety	MINI	Asia East	China	Adults with epilepsy who were Chinese citizens	unblinded	213	23.5	DSM 4	0.974	6	94	91.4
Sheng et al. (2010) [58]	HADS-A/anxiety	MINI	Asia East	China	Adult psychiatric outpatients	unblinded	70	25.5	DSM 4	0.805	6	86	79
Yang et al. (2014) [59]	HADS-A/anxiety	MINI	Asia East	China	Adult cardiac outpatients	unblinded*	100	15	DSM 4	0.81	6	81.6	75.8
Wang et al. (2017) [60]	HEI/Anxiety and depression	MINI	Asia East	China	Hospitalized patients aged 15+	unblinded*	763	7.11	DSM 4	0.88	11	88	76.6
Liu et al. (2008) [61]	PTSD screening tool/PTSD	DSM-IV PTSD criteria	Asia East	China	Survivors of a flood aged 16+	unblinded	27,267	9.5	DSM 4	0.858	3	87.9	97.9
Liu et al. (2007) [62]	PTSD screening tool/PTSD	DSM-IV PTSD criteria	Asia East	China	Child survivors of a flood aged 7–15	unblinded	6073	4.6	DSM 4	x	3	96.9	99
Ali et al. (1998) [63]	AKUADS/GAD and MDD	Interview	Asia South	Pakistan	Residents aged 16–60 in Karachi squatter settlement	unblinded	487	x	DSM 3	x	19	74	81
Kohrt et al. (2003) [64]	BAI/anxiety	DSM-IV criteria	Asia South	Nepal	Adults with psychiatric illness and controls	acceptable	363	Various†	DSM 4	x	14	91	89
Thapa et al. (2005) [65]	PCL-C/PTSD	CIDI	Asia South	Nepal	Adults residing in conflict areas	unblinded	290	53.4	DSM 4	0.81	50	80	80
Kohrt et al. (2011) [66]	CPSS/PTSD	K-SADS	Asia South	Nepal	Adolescents aged 11–14	good	162	6.4	DSM 4	0.77	20	68	73
Chaturvedi et al. (1994) [67]	HADS/anxiety	Interview	Asia South	India	Cancer patients of all ages	unblinded*	70	not specified	DSM 3	x	7	87	79
Ventevogel et al. (2007) [68]	HSCL/anxiety	PAS	Asia South	Afghanistan	Clinic patients aged 15+	good	116	24.1	x	0.61	2	75	43
Ventevogel et al. (2007) [68]	HSCL/depression and anxiety	PAS	Asia South	Afghanistan	Clinic patients aged 15+	good	116	24.1	x	0.61	2	69	67
Housen et al. (2018) [69]	HSCL-25/anxiety	MINI	Asia South	India	Adult general medical outpatients	good	290	3.5	DSM 4	0.81	1.75	73	81
Thapa et al. (2005) [65]	HSCL-25/anxiety	CIDI	Asia South	Nepal	Adults residing in conflict areas	unblinded	290	80.7	DSM 4	0.76	1.75	77	58
Ahmadi (2020) [70]	PHQ-4/anxiety	SCID	Asia South	Iran	Adults with coronary heart disease	unblinded*	279	not specified	DSM 5	0.94	7	80	94
Ahmadi (2020) [70]	PHQ-4/Anxiety and depression	SCID	Asia South	Iran	Adults with coronary heart disease	unblinded*	279	not specified	DSM 5	0.94	7	86	90
Russell et al. (2013) [71]	SCARED/anxiety	K-SADS-PL	Asia South	India	Adolescents aged 11–19	unblinded*	500	x	DSM 4	0.9	21	84.6	87.36
Namazi et al. (2013) [72]	UCLA PTSD (PTSD)	Interview	Asia South	Iran	Children aged 7–12 after earthquake	unblinded*	50	56	4-R	x	38	96	50
Tran et al. (2013) [73]	DASS-A/anxiety	SCID	Asia South East	Vietnam	Adult perinatal women	good	221	10.9	DSM 4	0.806	10	79.2	67
Sidik et al. (2012) [74]	GAD-7/anxiety	CIDI	Asia South East	Malaysia	Adult females	good	895	7.8	DSM 4	x	8	76	94
Yahya et al. (2015) [75]	HDRS/anxiety	DSM-IV	Asia South East	Malaysia	Patients with existing psychiatric disorder and controls	unblinded*	120	x	DSM 4	0.917	8	90	86.2
Silove et al. (2007) [76]	HTQ/PTSD	SCID	Asia South East	Thailand	Cambodian population in Thailand	good	118	20.3	DSM 4	0.71	2	63	61
Tran et al. (2019) [77]	K-10/anxiety	MINI-KID	Asia South East	Indonesia	Adolescents age 16–18	unblinded*	196	x	DSM 4	0.82	18	87.1	70.9
Tran et al. (2019) [77]	K-6/anxiety	MINI-KID	Asia South East	Indonesia	Adolescents age 16–19	unblinded*	196	x	DSM 4	0.8	12	83.9	73.3
Tran et al. (2011) [78]	Zung SAS/anxiety	Interview	Asia South East	Vietnam	Adult perinatal women	good	364	11.8	DSM 4	0.79	38	67.9	75.3
Tran et al. (2012) [79]	Zung SAS/anxiety	Interview	Asia South East	Vietnam	Men who are partners of pregnant or perinatal women	good	231	5.2	DSM 4	0.775	36	70.7	79
Mahfoud et al. (2011) [80]	AYMH/Anxiety and depression	Interview	Asia West	Lebanon	Socioeconomically disadvantaged children aged 10–14	good	153	17.6	DSM 4	0.71	39	63	79
Sawaya et al. (2016) [81]	GAD-7/anxiety	Interview	Asia West	Lebanon	Adult psychiatric outpatients	acceptable	176	x	DSM 4	0.57	10	57	53
Senturk et al. (2007) [82]	GHQ-12/anxiety	CIDI-PHCV	Asia West	Turkey	Adult leprosy patients	unblinded*	65	12.3	ICD 10	0.69	5	71	57
Malasi et al. (1991) [83]	HADS/anxiety	Interview	Asia West	Kuwait	Adult psychiatric outpatients and controls	acceptable	135	x	DSM 3		13	45	47
Senturk et al. (2007) [82]	HADS/anxiety	CIDI-PHCV	Asia West	Turkey	Adult leprosy patients	unblinded*	65	x	ICD 11	0.75	11	66	58
Yazici et al. (2018) [84]	PASS/anxiety	SCID-1	Asia West	Turkey	Adult women in perinatal period	unblinded*	312	19.2	DSM 4	0.94	16	95	84
Ibrahim et al. (2018) [85]	PCL-5/PTSD	DSM 5 interview	Asia West	Iraq	Adults living in a camp for displaced people in Iraq	good	206	37.75	DSM 5	0.82	23	82	70
Gormez et al. (2017) [86]	RCADS-GAD scale/GAD	K-SADS	Asia West	Turkey	Child psychiatry outpatients aged 8–17	unblinded*	483	not specified	DSM 4	x	7.5	70	71
Hariz et al. (2013) [87]	SCARED-C/anxiety	Interview	Asia West	Lebanon	Child and adolescent psychiatric patients	good	82	40.2	DSM 4	0.63	26	66	56
Hariz et al. (2013) [87]	SCARED-P/anxiety	Interview	Asia West	Lebanon	Child and adolescent psychiatric patients	good	82	x	DSM 4	0.7	24	67	55
Başoglu et al. (2001) [88]	TSSC/PTSD	CAPS	Asia West	Turkey	Survivors of 1999 August earthquake aged 16–70	acceptable	130	49	DSM 4	x	2	76	73
**Europe**													
Oruc et al. (2008) [89]	HTQ/(PTSD)	SCID	Europe Southern	Bosnia and Herzegovina	Adults enrolled in primary care clinic	very good	180	26	DSM 4	0.98	2.06	99.9	93.9
**South America**													
Zhong et al. (2015) [90]	GAD-7/GAD	CIDI	South America	Peru	Pregnant women aged 18–49 who speak Spanish	unblinded*	946	33.3	DSM 4	0.75	7	73.3	67.3
de Lima Osório et al. (2007) [91]	MINI-SPIN/Social anxiety disorder	SCID	South America	Brazil	University students	fair	2320	10.4	DSM 4	0.81	6	94	46
Gelaye et al. (2017) [92]	PCL-C/PTSD	CAPS	South America	Peru	Perinatal women	very good	3289	3	DSM 4	0.75	26	86	63
**Multiple Countries**													
Goldberg et al. (2017) [36]	PHC/current anxiety	CIS-R	South America, Asia South, Asia East, Central America	Brazil, Pakistan, China, Mexico	Primary care patients	fair	1488 (all countries)	Brazil: 26.5; Pakistan: 13; China: 18.9; Mexico: 23	ICD 11	0.77	3	75	68
**Meta-analyzed GAD-7 Values**													
Chibanda et al. (2016) [38] and Sawaya et al. (2016) [81]	GAD-7/anxiety	NA	NA	NA	NA	NA	NA	NA	NA	NA	≥10	76	64

**Quality**: ranges from highest to lowest (very good, good, fair, acceptable, unblinded, unblinded* (unblinded [unconfirmed so considered unblinded]); **x**: value not specified; **various†:** multiple values specified, see Appendix file; Abbreviations: *HADS* Hospital Anxiety and Depression Scale, *HADS-A* Hospital Anxiety and Depression Scale Anxiety subscale, *DASS* Depression Anxiety Stress Scales, *Zung SAS* Zung Self-Rating Anxiety Scale, *STAI* State Trait Anxiety Inventory, *EPDS* Edinburgh Postnatal Depression Scale, *HAM-A* Hamilton Anxiety Rating Scale, *K10/K6* Kessler 10/6, *GAD* Generalized Anxiety Test, *HDRS* Hamilton Depression Rating Scale, *HSCL* Hopkins Symptom Checklist, *MINI-SPIN* Mini-Social Phobia Inventory, *PHC* Primary Health Care Screening Tool, *GHC* General Health Questionnaire, *SCARED* Screen for Child Anxiety Related Disorders, *PASS* Perinatal Anxiety Screening Scale, *RCADS* Revised Children’s Anxiety and Depression Scales, *BAI* Beck Anxiety Inventory; *HTQ* Harvard Trauma Questionnaire, *PDS* Posttraumatic Diagnostic Scale, *PCL-C* PTSD Checklist-Clinician Version, *PHQ-4* Patient Health Questionnaire, *CPSS* Child PTSD Symptom Scale, *TSSC* Traumatic Stress Symptom Scale, *YSR* Youth Self-Report, *AKUADS* Aga Khan University Anxiety and Depression, *SRQ* Self-Reporting Questionnaire, *AYMH* Arab Youth Mental Health Scale, *HEI* Huaxi Emotional-Distress Index

## Discussion

This review aimed to examine the screening tools that have been validated to detect anxiety and PTSD in LMICs. The most commonly validated tools were the K-10 and GAD-7 for anxiety and the HTQ and the Posttraumatic Diagnostic Scale (PDS) for PTSD. It is difficult to recommend one screening tool for anxiety and PTSD respectively, as various tools and cut-off values were tested, and sensitivities and specificities varied based on region, country and screening tool. Indeed, only two studies tested the same tool using the same cut-off value and reported sufficient information to allow us to quantitatively synthesize the results. Locally validated screening tools for anxiety and PTSD need further evaluation in well-designed studies to assess whether they can improve the detection and management of these common disorders.

A total of 46 validated screening tools were found for anxiety disorders. The most common tool used to screen for anxiety disorders was the Kessler-10 followed by the GAD-7, which had wide ranges of sensitivities (57–94%) and specificities (53–94%) varying by region and sample size. While previously the HADS-A was recommended [23], our updated review found that it was not as widely validated as the GAD-7 and Kessler-10, although it had consistent specificities (72–79%) with a range of sensitivities (38–86%). The Kessler may have an added time-efficiency component, as it is possible to screen for multiple common mental disorders, whereas screening tools such as the HADS-A target anxiety specifically. The GAD-7 reported some of the highest sensitivities for detection of generalized anxiety disorder. Other anxiety disorders, including agoraphobia, panic disorder and social anxiety disorder were less commonly validated. Our results are consistent with a previous systematic review [23] and indicate using the GAD-7, K-10 or HAD-A yield good sensitivities and specificities while taking population-specific characteristics into account. Future research is needed to validate screening tools for these anxiety disorders in more regions.

The number of validations for PTSD increased from 10 to 19 since 2013 [23]. The HTQ and PDS were the most commonly validated tools for PTSD, and sensitivities were generally high. Our findings add that in addition to the previously recommended HTQ, the PDS should be considered in screening for PTSD [23]. Unfortunately, many tools were validated only once, preventing our combining them for analytic purposes. Only four PTSD validations describe children and adolescents, despite recent events that have displaced thousands of youth [93]. The prevalence of PTSD remains high in LMICs and is expected to rise given increasing civil unrest and war [19, 94]. The year 2018 saw the highest recorded number of displaced persons globally leading the authors to emphasize more attention into detection and treatment of PTSD [95].

Anxiety and depression had the fewest validations across our search [11] though were not the target of our validation given the existing literature on depression alone [23]. All tools with the exception of the HSCL-25 had only one validation. The only independently developed screening tool of all the studies was for anxiety and depression, developed in Zambia. These disorders commonly occur together, and further research is needed to determine which tools are best suited to a region’s mental health screening needs.

We searched four databases with a robust library of psychiatric publications available. We also placed minimal exclusion criteria on our searches so as to maximize the number of studies returned, and we additionally reviewed relevant systematic reviews for additional relevant papers. At every stage of the process from title/abstract screen to data abstraction, two reviewers assessed each article and numerical data point to reduce human error. Our search strategy and protocol were published in PROSPERO and were not altered from the time of submission, with the exception that we did not calculate diagnostic odds ratios (DORs), as they provide no guidance to clinicians on what screening tool and cut-off threshold would be most appropriate to use in clinical practice. Rather, we reported sensitivity and specificity of each screening tool and cutoff separately, to better describe the accuracies of individual tools and cut-offs.

Our extraction was limited by the individual papers’ specific data reporting. Varying prevalence of an individual study may affect the cut-off score, sensitivity and specificity of screening tools, and some studies did not publish prevalence. Providers should reference the prevalence of each specific disorder to ascertain whether the cut-off is applicable to their respective population. The majority of studies did not provide sensitivities and specificities for multiple cut-off values. Reporting multiple cut-off values and their respective sensitivity and specificity estimates would allow providers to decide which cut-off they would choose to optimize screening for their setting. A lower cut-off with a higher sensitivity may be desired if cases are not to be missed and false negatives reduced. A higher cut-off with a higher specificity may be desired if false positives are to be minimized. Furthermore, reporting multiple cut-off values and their respective sensitivity and specificity estimates would also allow researchers to better synthesize accuracy results across multiple studies in meta-analysis. In the present study, only two validations with identical cut-off scores for the GAD-7 could be combined via meta-analysis as no other validations of the same disorder with identical cut-off values provided sufficient information to conduct a meta-analysis (i.e., 2 × 2 table numbers). Studies used various versions of the DSM and ICD. While the symptomatology for psychiatric diagnoses have not changed significantly, providers should reference which version was used when conducting the validation of the screening tool (see Table 6).

Our review was also limited by the available publications on mental health screenings in LMICs. The entire region of Middle and North Africa, constituting over 300 million people, was not represented by a single validation while other regions such as South-East Asia were fairly well-represented. Cultural and linguistic factors may influence screening tool validation yet further discussion may be best served for individual validation papers. Most studies were rated in the lowest quality category of the modified Greenhalgh scale as they were unblinded, or downgraded to unblinded due to incomplete reporting. This is a severe limitation in the design of studies that may impact validation results; future studies should ensure adequate blinding in addition to the remainder of the quality checklist.

Our study did not look at CMDs or depression specifically, although we did consider anxiety and depression when screened for together. We chose to focus on anxiety and PTSD as they are less well-represented in the realm of LMIC validated screening tools. Additionally, anxiety and PTSD are becoming more important with the current displacement of millions of people due to civil unrest, socioeconomic upheaval and war.

The number of validated screening tools for mental health disorders as a whole has increased since 2013 [23]. However, no large increase in the number of validations for specific disorders was seen, and most screening tools from our search were validated only once. We advise researchers and providers to refer to Table 6 for a summary of validations for locations and disorders of interest and to use this table to identify their region of interest, find their disease focus of interest, and then identify what tools have been identified by the highest quality evidence.

## Conclusions

Mental health disorders are highly prevalent yet are frequently stigmatized and disregarded as medical diseases. Validated screening tools for anxiety and PTSD in LMIC have made considerable progress, with validations for both disorders almost doubling since the prior systematic review completed in December 2013 [23]. The increase in validated screening tools generally followed a regional pattern, with more emerging in countries already represented. For example, more tools have been validated in South Africa without an increase in validations in Botswana, Lesotho, Namibia or Swaziland. Middle and Northern Africa were also not well-represented by either anxiety or PTSD screening tools. The authors recognize that it may be near impossible to validate screening tools in areas of intense conflict and instability but acknowledge the need to evaluate screening tools in these areas.

The age distribution among screening tools was heavily biased towards the adult population. Children and adolescents accounted for only four of 19 validations for PTSD and six of 58 for anxiety and anxiety and depression. Given that age is skewed towards a younger population in LMICs [35], it is imperative that more research focuses on identifying anxiety and PTSD disorders in a pediatric population, especially in areas of increased civil war and conflict.

Use of brief screening instruments can bring much needed attention and research opportunities to various at-risk populations in LMICs. Many screening tools for anxiety and PTSD have been validated in LMICs, but there remain regions and subgroups of individuals for which more research is needed. Locally validated screening tools for anxiety and PTSD should be further evaluated in clinical trials to determine whether their use can reduce the burden of disease.

## Supplementary information

**Additional file 1.** Appendix

## Data Availability

All data generated or analysed during this study are included in this published article [and its supplementary information files].

## Abbreviations

PTSD: Post-traumatic stress disorder
LMICs: Low to middle income countries
CMDs: Common mental disorders
MINI and MINI-KID: Mini International Neuropsychiatric Interview
SCID, SCID-1 and NetSCID: Structured Clinical Interview for DSM
CIDI and CIDI-PHCV: Composite International Diagnostic Interview
CIS-R: Clinical Interview Schedule-Revised
PAS: Psychiatric Assessment Schedule
K-SADS and K-SADS-PL: Kiddie Schedule for Affective Disorders and Schizophrenia
CAPS and CAPS-5: Clinician-Administered PTSD Scale
AUC: Area under the curve
ROC: Receiver operating characteristic curve
DORs: Diagnostic odds ratios
HADS: Hospital Anxiety and Depression Scale
DASS: Depression Anxiety Stress Scales
Zung SAS: Zung Self-Rating Anxiety Scale
STAI: State Trait Anxiety Inventory
EPDS: Edinburgh Postnatal Depression Scale
HAM-A: Hamilton Anxiety Rating Scale
K10/K6: Kessler 10/6
GAD: Generalized Anxiety Test
HDRS: Hamilton Depression Rating Scale
HSCL: Hopkins Symptom Checklist
MINI-SPIN: Mini-Social Phobia Inventory
PHC: Primary Health Care Screening Tool
GHC: General Health Questionnaire
SCARED: Screen for Child Anxiety Related Disorders
PASS: Perinatal Anxiety Screening Scale
RCADS: Revised Children’s Anxiety and Depression Scales
BAI: Beck Anxiety Inventory
HTQ: Harvard Trauma Questionnaire
PDS: Posttraumatic Diagnostic Scale
PCL-C: PTSD Checklist-Clinician Version
CPSS: Child PTSD Symptom Scale
TSSC: Traumatic Stress Symptom Scale
CAPS: Clinician-Administered PTSD Scale
YSR: Youth Self-Report
AKUADS: Aga Khan University Anxiety and Depression
SRQ: Self-Reporting Questionnaire
AYMH: Arab Youth Mental Health Scale
HEI: Huaxi Emotional-Distress Index
